# Sine Spin flat detector CT can improve cerebral soft tissue imaging: a retrospective *in vivo* study

**DOI:** 10.1186/s41747-023-00412-2

**Published:** 2024-02-01

**Authors:** Niclas Schmitt, Lena Wucherpfennig, Jessica Jesser, Ulf Neuberger, Resul Güney, Martin Bendszus, Markus A. Möhlenbruch, Dominik F. Vollherbst

**Affiliations:** 1 Department of Neuroradiology, Heidelberg University Hospital, Im Neuenheimer Feld 400, 69120 Heidelberg, Germany; 2 Department of Diagnostic and Interventional Radiology, Heidelberg University Hospital, Im Neuenheimer Feld 420, 69120 Heidelberg, Germany

**Keywords:** Angiography, Ischemic stroke, Multidetector computed tomography, Neuroimaging, Thrombectomy

## Abstract

**Background:**

Flat detector computed tomography (FDCT) is frequently applied for periinterventional brain imaging within the angiography suite. Novel technical developments such as the Sine Spin FDCT (S-FDCT) may provide an improved cerebral soft tissue contrast. This study investigates the effect of S-FDCT on the differentiation between gray and white matter compared to conventional FDCT (C-FDCT) and multidetector computed tomography (MDCT).

**Methods:**

A retrospective analysis of a prospectively maintained patient database was performed, including patients who underwent mechanical thrombectomy in our institution and received S-FDCT or C-FDCT as well as MDCT. Differentiation between gray and white matter on the contralateral hemisphere to the ischemic stroke was analyzed quantitatively by contrast-to-noise ratio (CNR) and qualitatively (5-point ordinal scale).

**Results:**

In a cohort of 109 patients, MDCT demonstrated the best differentiation between gray and white matter compared to both FDCT techniques (*p* ≤ 0.001). Comparing both generations of FDCT, S-FDCT provided better visibility of the basal ganglia (*p* = 0.045) and the supratentorial cortex (*p* = 0.044) compared to C-FDCT both in quantitative and qualitative analyses. Median CNR were as follows: S-FDCT 2.41 (interquartile range [IQR] 1.66–3.21), C-FDCT 0.96 (0.46–1.70), MDCT 3.43 (2.83–4.17). For basal ganglia, median score and IQR were as follows: S-FDCT 2.00 (2.00–3.00), C-FDCT 1.50 (1.00–2.00), MDCT 5.00 (4.00–5.00).

**Conclusions:**

The novel S-FDCT improves the periinterventional imaging quality of cerebral soft tissue compared to C-FDCT. Thus, it may improve the diagnosis of complications within the angiography suite. MDCT provides the best option for x-ray-based imaging of the brain tissue.

**Relevance statement:**

Flat detector computed tomography is a promising technique for cerebral soft tissue imaging, while the novel Sine Spin flat detector computed tomography technique improves imaging quality compared to conventional flat detector computed tomography and thus may facilitate periinterventional diagnosis of gray and white matter.

**Key points:**

• Flat detector computed tomography (FDCT) is frequently applied for periinterventional brain imaging.

• The potential of novel Sine Spin FDCT (S-FDCT) is unknown so far.

• S-FDCT improves the visibility of cerebral soft tissue compared to conventional FDCT.

• Multidetector computed tomography is superior to both FDCT techniques.

• S-FDCT may facilitate the evaluation of brain parenchyma within the angiography suite.

**Graphical Abstract:**

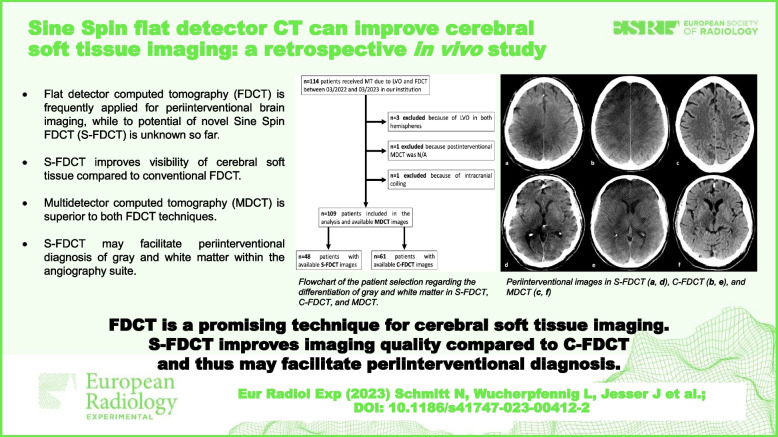

## Background

Flat detector computed tomography (FDCT) is a frequently applied imaging modality for periinterventional cerebral imaging [1–6]. Compared to multidetector computed tomography (MDCT), FDCT follows the idea of performing diagnostic and therapeutic imaging within the angiography suite to save crucial time [3, 7]. Since FDCT is frequently used for the detection of periinterventional complications, recent generations of FDCT promise an increased quality in cerebral imaging [1–3, 5–8].

The novel biplane C-arm system “ARTIS icono” (Siemens Healthineers, Erlangen, Germany), which features the latest generation of FDCT, the so-called syngo DynaCT Sine Spin (hereafter referred to as S-FDCT), was therefore primarily developed to improve cerebral soft tissue contrast [5]. During image acquisition, the S-FDCT adds a sinusoidal movement to its circular path, while the conventional FDCT (hereafter referred to as C-FDCT) follows a plain circular path. Moreover, S-FDCT acquires more projections than C-FDCT (546 *versus* 496 projections, respectively), resulting in a larger rotational coverage. A further innovation of S-FDCT is the utilization of a 4 × 4 binning instead of 2 × 2 in C-FDCT, which allows the acquisition of a smoother image [5, 9]. Detailed information on the technical acquisition protocols can be found in the “Methods” section of this paper.

Especially in emergency situations, such as acute ischemic stroke due to large vessel occlusion (LVO), FDCT constitutes a promising technique to estimate the Alberta Stroke Program Early CT Score (ASPECTS) within a “one-stop” management approach [7, 8, 10]. Recent publications investigating cerebral FDCT imaging quality focused *inter alia* on the detection of early ischemic lesions compared to MDCT [1, 2, 5, 6, 8].

Today, there is no systematic study available investigating cerebral soft tissue contrast regarding the differentiation of gray matter (GM) and white matter (WM) and thus the potential of S-FDCT compared to C-FDCT and MDCT. The differentiation between GM and WM and the technical capabilities of cerebral imaging of FDCT compared to MDCT are, however, essential to reliably detect pathologic changes, in particular ischemic lesions.

For this reason, the aim of the present study was the systematic comparison of the differentiation of GM and WM in supratentorial healthy brain parenchyma between S-FDCT, C-FDCT, and MDCT, and thus to explore the potential of S-FDCT for cerebral soft tissue imaging.

## Methods

### Patient selection

**Fig. 1:**
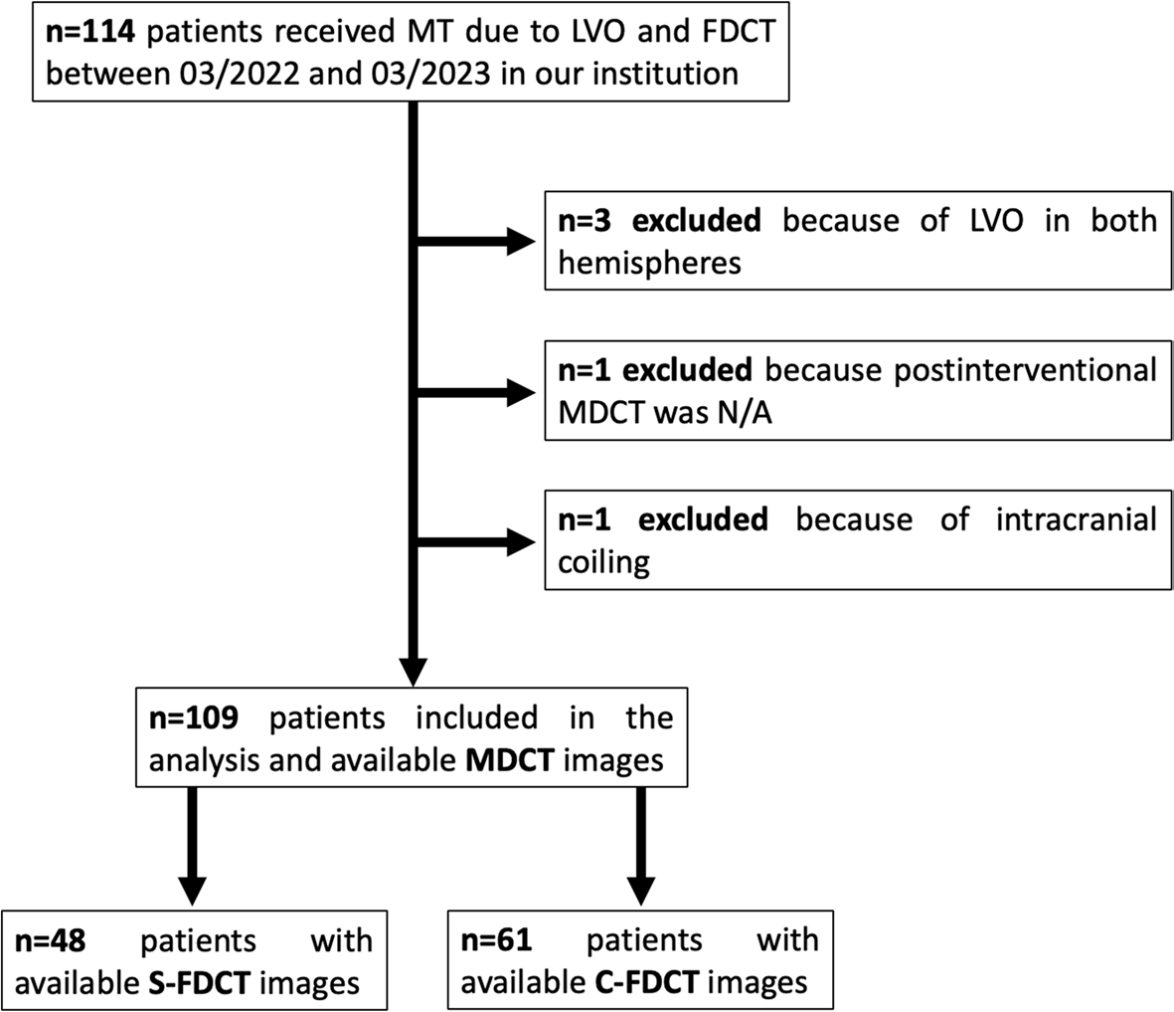
Flowchart illustrating the patient selection for the statistical analyses regarding the differentiation of gray and white matter in S-FDCT, C-FDCT, and MDCT. In total, 109 patients were eligible for this study, of whom 48 patients received S-FDCT and 61 patients received C-FDCT. *C-FDCT* Conventional flat detector computed tomography, *FDCT* Flat detector computed tomography, *LVO* Large vessel occlusion, *MDCT* Multidetector computed tomography, *MT* Mechanical thrombectomy, *N/A* Not available, *S-FDCT* Sine Spin flat detector computed tomography

A retrospective analysis of a prospectively maintained patient database was performed to identify all patients who underwent mechanical thrombectomy due to LVO between March 2022 and March 2023 (13 months) at our institution and received FDCT as well as MDCT immediately before (within 1 h) or within 24 h after mechanical thrombectomy. Patients with LVO of both hemispheres and intracranial metal devices (except intracranial stents because contralateral hemispheres were subjectively not affected by metal artifacts) were excluded from this study. A schematic overview of the patient selection is provided in Fig. 1. The study was conducted in accordance with the Declaration of Helsinki and its later amendments. Because of its retrospective character, additional written informed consent was waived by the local ethics committee.

### Imaging protocol

Non-contrast-enhanced image acquisition of all patients was performed on a biplanar angiography system (either on ARTIS icono or ARTIS Q, Siemens Healthineers, Erlangen, Germany) as well as on a MDCT system (SOMATOM Definition AS 64, Siemens Healthineers, Erlangen, Germany).

The following imaging parameters were applied for S-FDCT using the ARTIS icono angiography system (protocol “7sDCT Sine Spin”): 7-s rotational acquisition generating 546 projections with an angular step of 0.4° for a total coverage of 220° (110°/0° right anterior oblique (RAO) to 110°/0° left anterior oblique) with a pulse width of 4.0 ms, a tube voltage of 109 kVp, and a dose per frame of 1.82 μGy. Compared to C-FDCT, in which the craniocaudal angle stays at zero, the novel S-FDCT performs a slight craniocaudal modulation, like a sine curve, with an amplitude of 10° while scanning.

For C-FDCT, the following imaging parameters were applied (protocol “20sDCT Head”) using the ARTIS Q angiography system: 20-s rotational acquisition generating 496 projections with an angular step of 0.4° for a total coverage of 200° (100°/0° right anterior oblique to 100°/0° left anterior oblique) with a pulse width of 12.5 ms, a tube voltage of 109 kVp, and a dose per frame of 1.82 μGy.

MDCT imaging using the SOMATOM Definition AS 64 was performed with standard settings according to our clinical routine with a tube voltage of 120 kVp, a tube current of 20 mAs, and a J40s kernel for image reconstruction. Moreover, Sinogram Affirmed Iterative Reconstruction, SAFIRE, was applied to all MDCT images, while no iterative reconstruction method was utilized for S-FDCT and C-FDCT images. All images of S-FDCT, C-FDCT, and MDCT were reconstructed with a slice thickness of 4 mm for final analyses.

### Quantitative image analysis

Quantitative image analysis regarding the differentiation of GM and WM in both FDCT systems and MDCT was conducted on a picture archiving and communication workstation (CENTRICITY PACS 4.0; General Electric Healthcare, Barrington, IL, USA). Therefore, two similar regions of interest (ROI), each with a circular configuration and a diameter of 5 mm, were drawn manually in the center of the lentiform nucleus and the center of the supraventricular WM on the contralateral hemisphere to the ischemic stroke. In patients with occlusion of the basilar artery, ROIs were drawn on the left hemisphere. For both ROIs, the mean density units in FDCT and mean Hounsfield units in MDCT as well as the corresponding standard deviations were calculated. The manual drawing of each ROI was performed in consensus with a neuroradiology resident and a neuroradiology attending (seven years and ten years of experience in diagnostic imaging, respectively). In the next step, the contrast-to-noise ratio (CNR) of each patient’s FDCT and MDCT dataset was determined using the following formula:

$$\mathrm{CNR}=\frac{|\mathrm{Mean \,DU}/\mathrm{HU}_{\mathrm{GM}}-\mathrm{ Mean \,DU}/\mathrm{HU}_{\mathrm{WM}}|}{\mathrm{Standard\, deviation}_{\mathrm{\,WM}}}$$

**Fig. 2:**
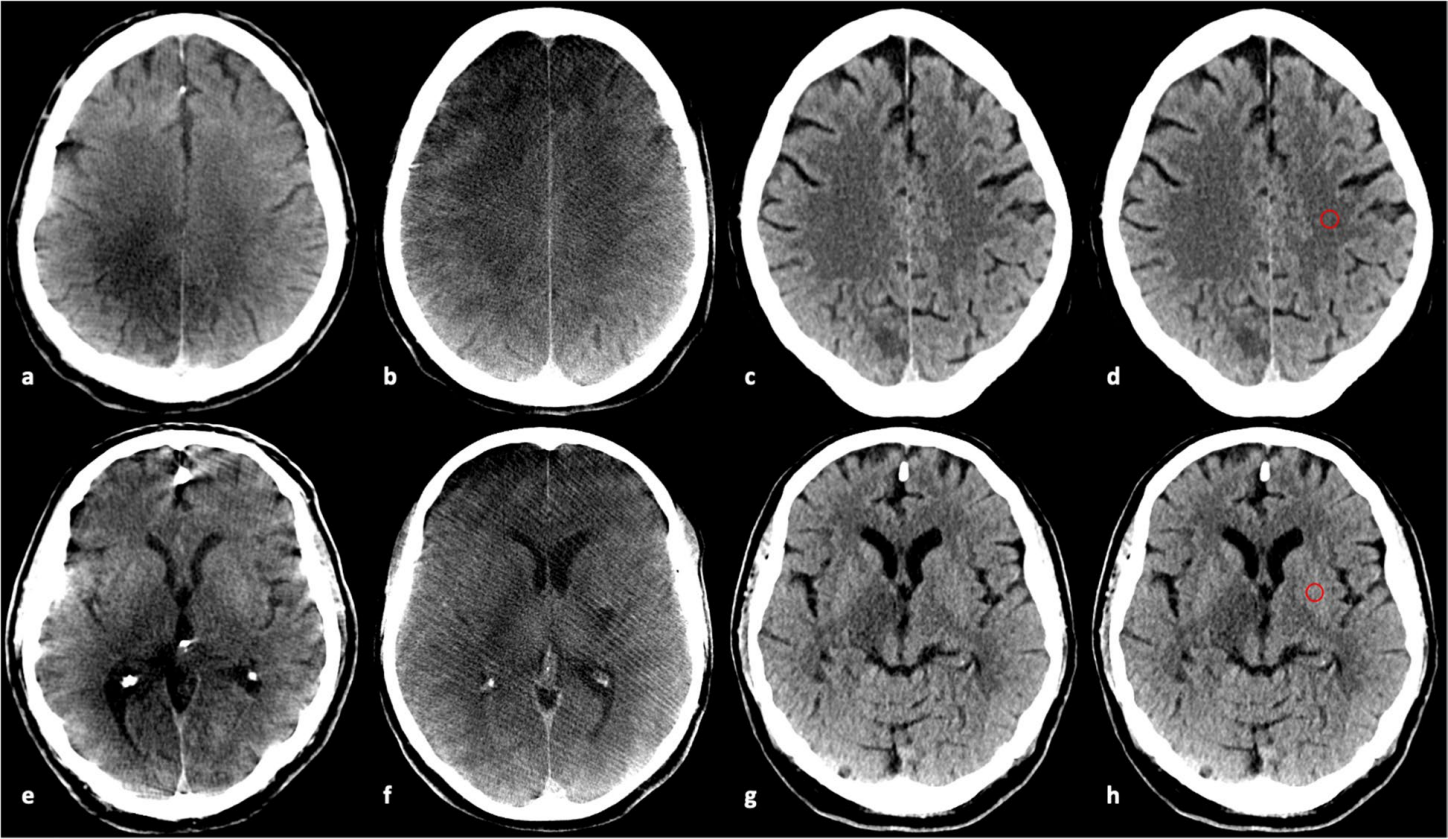
Illustration of periinterventional images in S-FDCT (**a**, **e**), C-FDCT (**b**, **f**), and MDCT (**c**, **g**) for the supraventricular white matter (**a**–**d**) and the basal ganglia (**e**–**h**). Moreover, representative images of the process of quantitative analysis are shown (**d**, **h**). Therefore, a standardized region of interest of 5 mm in diameter was drawn manually into the center of the lentiform nucleus (**h**) and the supraventricular white matter (**d**). *C-FDCT* Conventional flat detector computed tomography, *MDCT* Multidetector computed tomography, *S-FDCT* Sine Spin flat detector computed tomography

A schematic illustration of the process of quantitative analysis is provided in Fig. 2.

### Qualitative image analysis

Qualitative analysis of the FDCT and MDCT images was performed on a CENTRICITY Picture Archiving and Communication System 4.0 workstation (General Electric Healthcare, Barrington, IL, USA) by two different readers (Reader 1 and Reader 2), both with seven years of experience in diagnostic imaging. Both readers were blinded to the type of FDCT and MDCT, while manual adjustment of the window width and window level was allowed. The visibility and thus the differentiation of the basal ganglia and the supratentorial cortex were graded separately by using a 5-point ordinal scale: (1) no visibility—non-diagnostic; (2) poor visibility—limitedly diagnostic; (3) moderate visibility—partly diagnostic; (4) good visibility—sufficiently diagnostic; and (5) excellent visibility—fully diagnostic.

### Statistics

Statistical analysis was performed using the GraphPad Prism software (version 9.5.1, San Diego, CA, USA). The assessment of the inter-reader agreement was conducted by calculating the Cohen’s *κ* coefficient, which was interpreted as follows: $\le$ 0.20, poor agreement; 0.21–0.40, fair agreement; 0.41–0.60, moderate agreement; 0.61–0.80, good agreement; and 0.81–1.00, very good agreement [11, 12]. To evaluate statistical differences between the study groups, the Kruskal–Wallis test was conducted. Post hoc, Dunn’s test for multiple comparisons using statistical hypothesis testing was performed to assess differences between S-FDCT, C-FDCT, and MDCT regarding the differentiation of GM and WM as well as the radiation doses. Moreover, the Shapiro–Wilk test was used to test for normality. Results of the quantitative analysis are presented as median CNR and interquartile range (IQR) and of the qualitative analysis as the median score and IQR. A further Mann–Whitney *U* test was performed to evaluate statistical differences in the age of both study groups (S-FDCT and C-FDCT). The level of statistical significance was defined as *p* < 0.05.

## Results

A total of 114 patients underwent mechanical thrombectomy due to LVO and received FDCT between March 01, 2022, and March 01, 2023 (13 months) in our institution. Of this cohort, an overall number of 109 patients (age 74.5 ± 11.4 years [mean ± standard deviation]; 56 females, 51% and 53 males, 49%) were eligible for the present study. Five patients were excluded because of the following criteria: LVO in both hemispheres (*n* = 3); periinterventional MDCT not available because MRI was performed (*n* = 1); and periprocedural coiling of the cerebral arteries performed (*n* = 1). Of the 109 eligible patients, 48 received S-FDCT (44%; aged 72.8 ± 11.1 years; 25 females and 23 males) and 61 received C-FDCT (56%; aged 75.7 ± 11.5 years; 30 females and 31 males). No statistical difference in the age of both study groups was found (*p* = 0.125). An overview of the patient selection is given in Fig. 1.

**Fig. 3:**
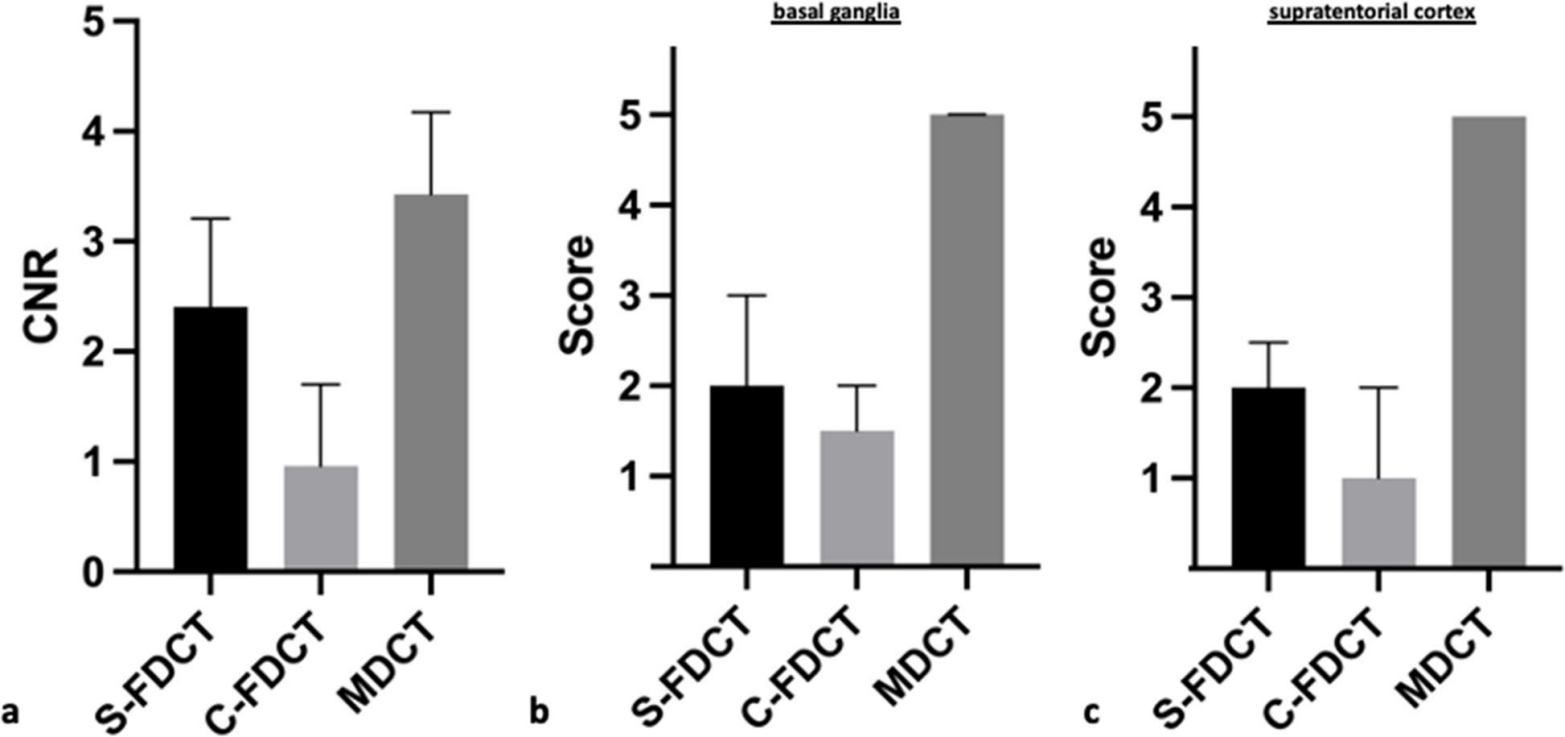
At quantitative analysis (**a**) the lentiform nucleus of the contralateral hemisphere to the ischemic stroke demonstrated a higher CNR and thus a better differentiation of gray matter (GM) for S-FDCT compared to C-FDCT. Similar results were found in the qualitative analyses with a better differentiation of the basal ganglia (**b**) and the supratentorial cortex (**c**) for S-FDCT compared to C-FDCT. MDCT provided the best visibility of GM in both analyses. Bars = median; whiskers = interquartile range. *C-FDCT* Conventional flat detector computed tomography; *CNR* Contrast-to-noise ratio; *MDCT* Multidetector computed tomography; *S-FDCT* Sine Spin flat detector computed tomography

Examples of S-FDCT, C-FDCT, and MDCT images are shown in Fig. 2. The results of the quantitative image analysis regarding the quality and thus the differentiation of GM and WM are summarized in Fig. 3 and Table 1. Normality test for CNR revealed normal distribution of S-FDCT data, while CNR data of C-FDCT and MDCT were distributed not normally. The Kruskal–Wallis test demonstrated a significant difference between the individual study groups (*p* < 0.001). In accordance with the post hoc test as well as the descriptive analysis, there was a higher CNR and thus a better differentiation of the lentiform nucleus in S-FDCT compared to C-FDCT, while CNR showed the highest values for MDCT (S-FDCT 2.41 (1.663.21) *versus* C-FDCT 0.96 (0.46–1.70) *versus* MDCT 3.43 (2.83–4.17), *p* < 0.001 for all).

**Table 1:** Summary of the results of the quantitative and qualitative analyses

**Quantitative analysis (CNR)**					
S-FDCT		C-FDCT			MDCT
2.41 (1.66–3.21)		0.96 (0.46–1.70)			3.43 (2.83–4.17)
*versus* C-FDCT	*p* < 0.001	*versus* MDCT	*p* < 0.001		
*versus* MDCT	*p* < 0.001				
**Qualitative analysis (1-to-5 score)**					
	S-FDCT		C-FDCT		MDCT
Basal ganglia	2.00 (2.00–3.00)		1.50 (1.00–2.00)		5.00 (4.00–5.00)
	*versus* C-FDCT	*p* = 0.045	*versus* MDCT	*p* < 0.001	
	*versus* MDCT	*p* < 0.001			
Supratentorial cortex	2.00 (1.66–2.50)		1.00 (1.00–2.00)		5.00 (5.00–5.00)
	*versus* C-FDCT	*p* = 0.044	*versus* MDCT	*p* < 0.001	
	*versus* MDCT	*p* < 0.001			

Results are provided as median and interquartile range of the CNR (quantitative analysis) and the score on the five-point scale (qualitative analysis). *C-FDCT* Conventional flat detector, *CNR* Contrast-to-noise ratio, *MDCT* Multidetector computed tomography, *S-FDCT* Sine Spin flat detector CT

The results of the qualitative analysis are summarized in Fig. 3 and Table 1. The inter-reader reliability showed an overall very good agreement (*κ* = 0.829; range 0.788–0.871), while the normality test revealed no normal distribution of the qualitative data. Regarding the differentiation of GM and WM for the basal ganglia and the supratentorial cortex, the Kruskal–Wallis test demonstrated statistical differences (*p* < 0.001). There was better visibility of the GM for S-FDCT compared to C-FDCT (basal ganglia, *p* = 0.045; supratentorial cortex, *p* = 0.044), while both were best visible in MDCT (basal ganglia, S-FDCT 2.00 (2.00–3.00) *versus* C-FDCT 1.50 (1.00–2.00) *versus* MDCT 5.00 (4.00–5.00), *p* < 0.001; supratentorial cortex, S-FDCT 2.00 (1.66–2.50) *versus* C-FDCT 1.00 (1.00–2.00) *versus* MDCT 5.00 (5.00–5.00), *p* < 0.001).

Radiation doses were statistically different between all study groups (*p* < 0.001) with the highest delivered radiation doses for S-FDCT (median 196.4 mGy, range 181.1–203.4). Radiation dose data for S-FDCT and MDCT were not distributed normally, while the corresponding C-FDCT data was distributed normally. Detailed information on the radiation doses and statistical differences can be found in Table 2.

**Table 2:** Summary of the radiation doses

S-FDCT		C-FDCT		MDCT
196.4 (181.1–203.4) mGy		165.0 (148.5–183.5) mGy		37.4 (35.6–40.4) mGy
versus C-FDCT	*p* = 0.039	*versus* MDCT	*p* < 0.001	
*versus* MDCT	*p* > 0.001			
*versus* MDCT	*p* > 0.001			

Data on the radiation doses of the different x-ray-based imaging techniques and statistical differences among them. Results are provided as median and interquartile range. *C-FDCT* Conventional flat detector computed tomography, *MDCT* Multidetector computed tomography, *S-FDCT* Sine Spin flat detector computed tomography

## Discussion

FDCT is an increasingly applied method for periinterventional imaging [1–6]. Therefore, novel generations of angiography systems such as the biplane C-arm system ARTIS icono have been developed to provide an improved cerebral soft tissue visualization [5]. In order to reliably detect early changes of ischemic infarction, visualization of healthy brain parenchyma and especially the differentiation of GM and WM are essential. However, there are currently no studies available investigating and comparing cerebral soft tissue contrast in healthy brain parenchyma in different generations of FDCT and MDCT and thus the potential benefit of S-FDCT. Results of the present study demonstrate an enhanced visibility with an improved differentiation of GM in contrast to WM for S-FDCT compared to C-FDCT in qualitative and quantitative analyses. Both analyses further demonstrated the best brain tissue visibility for MDCT.

In recent years, several studies investigated the potential of FDCT for the detection of ischemic brain lesions [1–3, 5–8]. Therefore, most studies focused on a comparison of FDCT within a “one-stop” management approach in patients with LVO compared to MDCT as a first step working as a gatekeeper to interventional angiography [7, 8]. Maier et al. [2] compared FDCT and MDCT in 25 patients with acute ischemic stroke to assess their baseline ASPECTS with no difference, using the ARTIS Q angiography system for FDCT image acquisition. However, they described the tendency of a better imaging quality in MDCT. Lehye et al. [1] described similar results for the ASPECTS rating in a cohort of 102 patients, whereas our analyses for a total of 109 patients found a statistically significant better visibility of the GM in MDCT compared to both generations of FDCT. The difference might be partly explained by the varying patient cohorts as well as the applied MDCT scanners of the different studies. However, our analyses included a further quantitative evaluation, calculating the CNR of the lentiform nucleus in contrast to the WM, whereas most studies only carried out a qualitative evaluation. Petroulia et al. [5] provided the first trial, using S-FDCT of the ARTIS icono angiography suite for soft tissue image acquisition. Including 49 patients, they described a better differentiation of GM and WM for S-FDCT compared to MDCT, which is in line with our findings.

Comparing S-FDCT and C-FDCT for the first time within the present study, S-FDCT demonstrated an enhanced differentiation of the GM and WM at quantitative and qualitative analyses, while S-FDCT images resulted to be at least partly diagnostic and C-FDCT images were limitedly diagnostic. In both analyses, MDCT featured the best results and was therefore superior to both FDCT techniques. From a technical point of view, the enhanced visibility of the GM in S-FDCT compared to C-FDCT might be achieved by the increased number of 546 projections and the greater rotational coverage of 220° in combination with the craniocaudal sine modulation. It can be assumed that these technical differences and especially the sine modulation of the C-arm allow a more complete scan of the brain with less artifacts emerging from surrounding structures, such as the skull, and thus providing an enhanced soft tissue quality [9].

Transferring these findings to clinical routine, S-FDCT provides advantages compared to C-FDCT for periinterventional cerebral soft tissue imaging, coming at the price of an increased radiation dose. Comparing both FDCT techniques to MDCT, the multidetector technique accompanies a significantly reduced dose of radiation and is therefore not only in terms of image quality but also in relation of radiation exposure superior to the FDCT techniques. Nevertheless, the enhanced soft-tissue contrast seems to be subjectively still limited with a questionable feasibility regarding early changes in acute stroke diagnostics.

We acknowledge that this study has several limitations. First, this study was performed in a single-center situation with a retrospective design. Including different patients in a multicenter trial using different MDCT scanners would provide a more heterogenous database and might have an influence on the findings. Second, the time difference between MDCT and FDCT image acquisition, as well as the intravascular residues of contrast media if the FDCT images were acquired postprocedurally, may affect the differentiation of GM and WM. Third, only the supratentorial cortex was analyzed since relevant ischemic changes primarily affect the basal ganglia in CT. All ROIs for the quantitative analysis were drawn manually which might lead to certain bias, especially in regions adjacent to the skull with varying bony artifacts. Furthermore, the assessment was conducted in brain tissue contralateral to the LVO with a potential impact on the underlying disease.

In conclusion, FDCT constitutes a promising technique for periprocedural cerebral soft tissue imaging. Compared to the C-FDCT generations, the novel S-FDCT improves the periinterventional imaging quality of brain tissue and thus may improve a reliable evaluation of the gray and white matter, such as the assessment of infarcted brain parenchyma within the angiography suite.

## Data Availability

All data generated or analyzed during this study are included in this published article.

## Abbreviations

ASPECTS: Alberta Stroke Program Early CT Score
C-FDCT: Conventional flat detector computed tomography
CNR: Contrast-to-noise ratio
FDCT: Flat detector computed tomography
GM: Gray matter
IQR: Interquartile range
LVO: Large vessel occlusion
MDCT: Multidetector computed tomography
ROI: Region of interest
S-FDCT: Sine Spin flat detector computed tomography
WM: White matter
