# Correction: Sine Spin flat detector CT can improve cerebral soft tissue imaging: a retrospective in vivo study

**DOI:** 10.1186/s41747-026-00773-4

**Published:** 2026-07-16

**Authors:** Niclas Schmitt, Lena Wucherpfennig, Jessica Jesser, Ulf Neuberger, Resul Güney, Martin Bendszus, Markus A. Möhlenbruch, Dominik F. Vollherbst

**Affiliations:** 1 Department of Neuroradiology, Heidelberg University Hospital, Heidelberg, Germany; 2 Department of Diagnostic and Interventional Radiology, Heidelberg University Hospital, Heidelberg, Germany

**Correction to:** **European Radiology Experimental**

10.1186/s41747-023-00412-2, published online 01 February 2024

In the sentence beginning with “The following imaging parameters were applied...” under “Imaging protocol” section, “a tube voltage of 119 kVp” should have read “a tube voltage of 109 kVp”.

The original article has been corrected.

